# Caffeine Neuroprotective Mechanism Against β-Amyloid Neurotoxicity in SHSY5Y Cell Line: Involvement of Adenosine, Ryanodine, and N-Methyl-D-Aspartate Receptors

**DOI:** 10.15171/apb.2017.069

**Published:** 2017-12-31

**Authors:** Mojtaba Keshavarz, Majid Reza Farrokhi, Atena Amiri

**Affiliations:** Shiraz Neuroscience Research Center, Shiraz University of Medical Sciences, Shiraz, Iran.

**Keywords:** Caffeine, N-methyl-D-Aspartate, Adenosine, Dantrolene, β-amyloid

## Abstract

***Purpose:*** Some reports have shown neuroprotective effects of caffeine in several neurodegenerative disorders. However, its mechanism of action is not completely clear. Therefore, the aim of this study was to explore the interference of ryanodine, N-methyl-D-aspartate (NMDA) and adenosine modulators with the neuroprotective effects of caffeine against β-amyloid (Aβ) neurotoxicity in the SHSY5Y cells.

***Methods:*** The SHSY5Y cells were treated with Aβ23-35 (20µM) and/or caffeine (0.6 and 1mM), or both for 24 hours. Adenosine (20, 40, 60, 80, 100µM), NMDA (20, 50, 70, 90µM), dantrolene (2, 4, 6, 8, 10µM) were also added to the medium and incubated for 24 hours. The cell viability was measured via the MTT (3-[4,5-dimethylthiazol-2-yl]-2,5-diphenyl tetrazolium bromide) method. The data were analyzed using one-way ANOVA followed by Bonferroni test.

***Results:*** Caffeine at all the used concentrations (0.6, 0.8, 0.9, 1, and 3mM) significantly protected neuronal cells against Aβ neurotoxicity. Adenosine at the concentrations of 20, 40, 80 and 100μM diminished the neuroprotective effects of caffeine (0.6 and 1mM) against Aβ neurotoxicity. NMDA at the concentrations of 20, 50, 70 and 90μM blocked caffeine (0.6 and 1mM) neuroprotective effects. Dantrolene at the concentration of 2, 4, 6, 8 and 10μM diminished the neuroprotective effects of caffeine (0.6mM) and at the concentrations of 2 and 10μM impede caffeine (1mM) neuroprotection against Aβ neurotoxicity.

***Conclusion:*** Caffeine produced neuroprotective effect against Aβ neurotoxicity. Blockade of adenosine and NMDA receptors, as well as the activation of ryanodine receptors, may contribute to the neuroprotective effects of caffeine.

## Introduction

Extracellular aggregation of β-amyloid peptides (Aβ) and hyperphosphorylated tau protein (neurofibrillary tangles) may be the most important causes of neural degeneration in Alzheimer’s disease (AD).^1^ Moreover, accumulating data have implied that deregulated calcium signaling may have an important contribution to the neural cell death in AD.^2^ Interestingly, altered intracellular calcium homeostasis emerges earlier than neuropathological abnormalities observed in AD.^3^

Calcium is an important signal transduction molecule^4^ which is involved in a wide range of neural functions like cell growth, differentiation, metabolism, exocytosis, and apoptosis.^4^ Several neuronal systems, including N-methyl-D-Aspartate (NMDA), adenosine and ryanodine receptors maintain the intracellular concentration of calcium within a narrow normal range.^5-7^ On the other hand, ryanodine, NMDA and adenosine receptors have possible roles in the pathophysiology and treatment of AD.^8-10^ Thus, these receptors may be the targets of neuroprotective agents in AD.

Caffeine is the most popular psychoactive drug around the world^11^ with important modulatory effects on intracellular calcium in the central nervous system (CNS).^5,12^ Caffeine effects may be related to the non-specific modulation of several systems in the CNS. At the normal daily consumption (2.4 to 4.0 mg/kg or 2 to 4 cups of coffee per person),^11^ the primary target of caffeine is the non-selective antagonism of adenosine receptors.^13^ Ryanodine receptors are the other physiological targets of caffeine in the CNS which mediates caffeine-induced intracellular calcium release.^14^ In contrast, caffeine effects on the NMDA receptors are not fully elucidated.

Recent evidence has shown that caffeine exerts profound effects on motor, behavior, information processing, and cognitive performance.^15^ It has been also demonstrated that caffeine can produce neuroprotective effects in different neurodegenerative diseases.^16^ In-vivo and in-vitro studies have also shown that caffeine or coffee has protective effects against AD.^17^ However, the exact mechanism of neuroprotective effects of caffeine in AD is not completely clear. Therefore, the aim of this study was to explore the interference of ryanodine, NMDA and adenosine modulators with the neuroprotective effects of caffeine against Aβ neurotoxicity in the SHSY5Y cell line.

## Materials and Methods

### Materials

Human SHSY5Y cells were purchased from Pasteur Institute (Tehran, Iran). Cell culture materials including DMEM/F12, FBS (fetal bovine serum), and Penicillin-Streptomycin were obtained from Gibco^®^life technologies^™^ (New York, USA). Aβ25-35, caffeine, dantrolene sodium salt, NMDA, and adenosine were purchased from Sigma-Aldrich (St. Louis, USA).

### Neuronal Cell Culture 

The human SHSY5Y cells were grown in DMEM/F12 (1:1) media supplemented with 10 % fetal bovine serum, 100U/ml penicillin, and 100µg/ml streptomycin. The cells were seeded at a density of 10^5^ cells/well in the 96-well plates for the MTT (3-[4,5-dimethylthiazol-2-yl]-2,5-diphenyl tetrazolium bromide) experiments. The plates were maintained at 37°C in 95 % humidified atmosphere (air) with 5 % CO_2_. After 24 hr of incubation, the cells were treated with Aβ and/or other agents.

### Aβ25–35 Preparation

Aβ25–35 was dissolved in sterile distilled water at a concentration of 2μg/μl and kept at −70 °C until use. For the aggregation process, Aβ25–35 was incubated at 37 °C for 4 days before the administration in the cell culture.

### Treatment

Caffeine, dantrolene (a ryanodine receptor antagonist), adenosine and NMDA were dissolved in PBS (Phosphate Buffered Solutions). The effective concentration of Aβ25-35 and caffeine was obtained through dose response experiments and MTT assay. On the day of treatment, the serum-free medium was treated with Aβ23-35 (20µM, selected according to pilot studies) and/or caffeine (0.6 and 1mM, selected according to pilot studies), or both for 24 hours. Adenosine (20, 40, 60, 80, 100µM), NMDA (20, 50, 70, 90µM), dantrolene (2, 4, 6, 8, 10µM) were also added to the medium and incubated for 24 hours.

### Cell Viability Assay

Twenty-four hours after treatments, 5 mg/ml MTT reagent was added to the cell culture media. Four hours later, the media was gently removed and the precipitations in each well were dissolved in 100 µl of DMSO. We measured the absorbance at 570 nm using a microplate reader (Synergy HT, Biotek®) as an index of neuronal viability.

### Statistical analysis

The variables were analyzed using one-way ANOVA followed by Bonferroni test. The significance level was considered as <0.05. All analyses were performed suing SPSS software version 23.

## Results and Discussion

Aβ at a concentration of 20μM decreased about 71% in the neural viability in the SY-SY5Y cell lines compared with the control group. Moreover, our analysis showed that different concentrations of caffeine significantly protected neuronal cells against Aβ neurotoxicity (F(df)= 16.81(6), p= 0.000). We chose two concentration of caffeine (0.6 and 1 mM) to evaluate the interaction of adenosine, NMDA, and dantrolene with caffeine neuroprotective effects.

**Figure 1:**
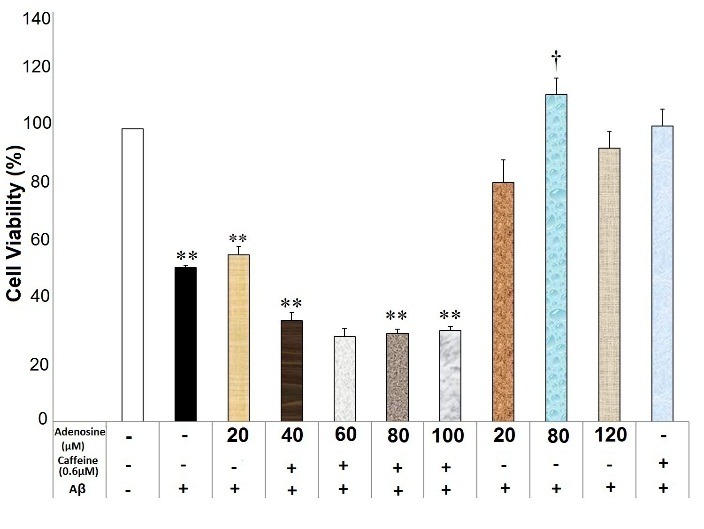
Adenosine affected caffeine (0.6mM) neuroprotective effects against Aβ neurotoxicity in the SHSY5Y neuroblastoma cells. Data are the mean + standard error of four experiments and were analyzed using one-way ANOVA followed by Bonferroni test. ** shows the significance at 0.001 compared with the β-amyloid (Aβ) + caffeine (0.6mM)-treated group, and † significance at 0.05 compared with the Aβ-treated group. Cell viability was assessed via MTT (3-[4,5-dimethylthiazol-2-yl]-2,5-diphenyl tetrazolium bromide) test.

**Figure 2:**
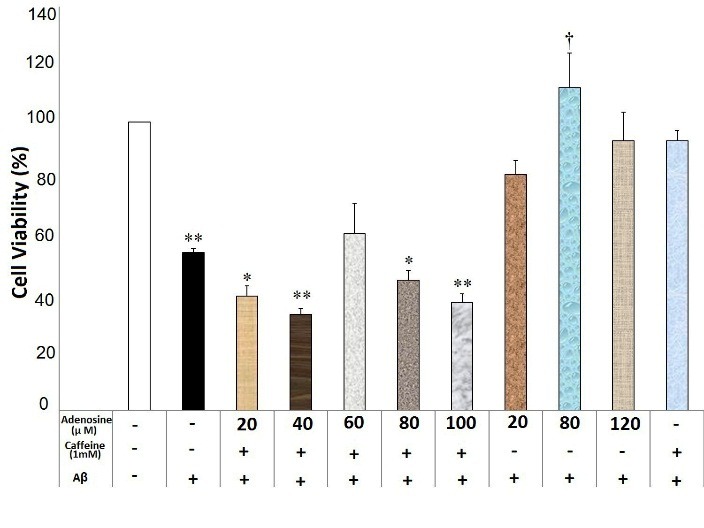
Adenosine affected caffeine (1mM) neuroprotective effects against Aβ neurotoxicity in the SHSY5Y neuroblastoma cells. Data are the mean + standard error of four experiments and were analyzed using one-way ANOVA followed by Bonferroni test. * and ** shows the significance at 0.05 and 0.001 compared with the β-amyloid (Aβ) + caffeine (1mM)-treated group, respectively, and † significance at 0.05 compared with the Aβ-treated group. Cell viability was assessed via MTT (3-[4,5-dimethylthiazol-2-yl]-2,5-diphenyl tetrazolium bromide) test.

Adenosine at the concentrations of 20, 40, 60, 80 and 100μM diminished the neuroprotective effects of caffeine (0.6mM) against Aβ neurotoxicity (F(9)= 21.35, p=0.000) (Figure 1). Moreover, adenosine at the concentrations of 20, 40, 80 and 100μM blocked caffeine (1mM) neuroprotective effects against Aβ neurotoxicity (F(9)= 8.77, p=0.000) (Figure 2). There was a significant difference between different concentrations of adenosine (without caffeine) against Aβ neurotoxicity (F(3)=4.902, p=0.019). The pairwise comparison showed that adenosine only at a concentration of 80μM reduced Aβ neurotoxicity compared with the Aβ-treated group (p=0.036).

**Figure 3:**
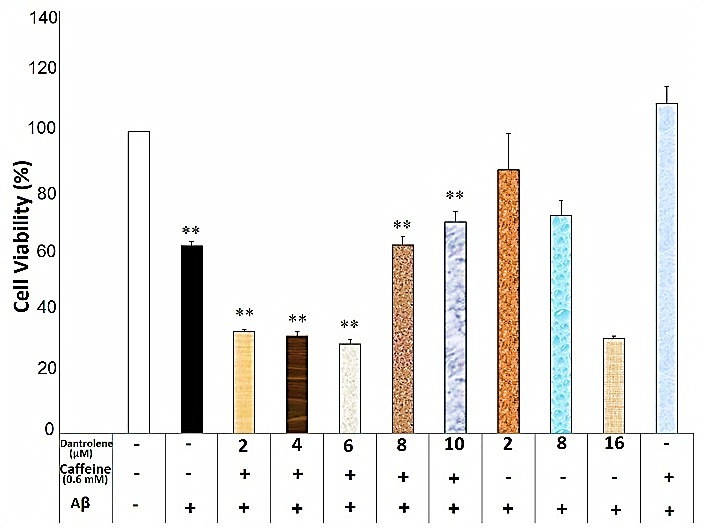
Dantrolene affected caffeine (0.6mM) neuroprotective effects against Aβ neurotoxicity in the SHSY5Y neuroblastoma cells. Data are the mean + standard error of four experiments and were analyzed using one-way ANOVA followed by Bonferroni test. ** shows the significance at 0.001 compared with the β-amyloid (Aβ) + caffeine (0.6mM)-treated group. Cell viability was assessed via MTT (3-[4,5-dimethylthiazol-2-yl]-2,5-diphenyl tetrazolium bromide) test.

**Figure 4:**
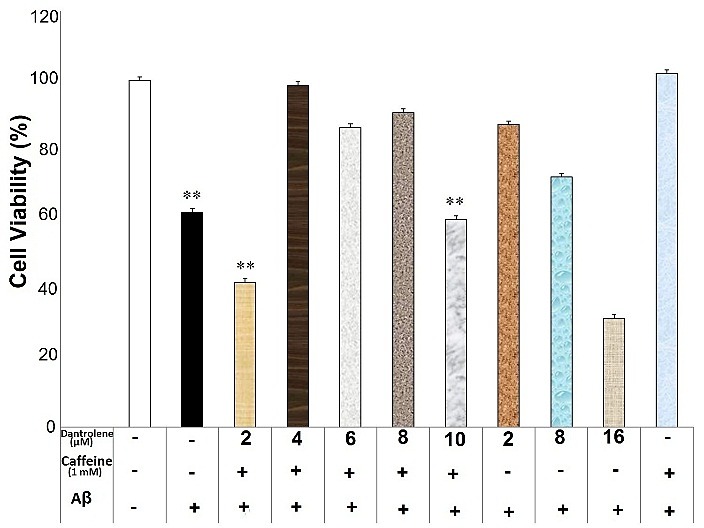
Dantrolene affected caffeine (1mM) neuroprotective effects against Aβ neurotoxicity in the SHSY5Y neuroblastoma cells. Data are the mean + standard error of four experiments and were analyzed using one-way ANOVA followed by Bonferroni test.
** shows the significance at 0.001 compared with the β-amyloid (Aβ) + caffeine (1mM)-treated group. Cell viability was assessed via MTT (3-[4,5-dimethylthiazol-2-yl]-2,5-diphenyl tetrazolium bromide) test.

Dantrolene at the concentration of 2, 4, 6, 8 and 10μM diminished the neuroprotective effects of caffeine (0.6mM) against Aβ neurotoxicity (F(7)= 43.75, p=0.000) (Figure 3). Furthermore, dantrolene at the concentrations of 2 and 10μM impeded the caffeine (1mM) neuroprotection against Aβ neurotoxicity (F(7)=16.26, p=0.000)(Figure 4). In contrast, dantrolene at the concentrations of 4, 6 and 8μM had no effect on the caffeine (1mM) neuroprotection (Figure 4). There was a significant difference between the different concentration of dantrolene (without caffeine use) against Aβ neurotoxicity (F(4)=8.81, p=0.001), though the pairwise comparison showed no significant difference between each concentration of dantrolene and the Aβ-treated group (p>0.05).

**Figure 5:**
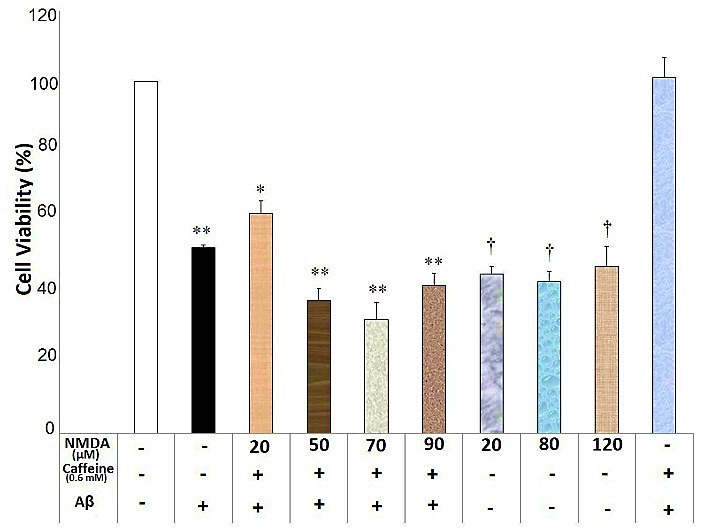
N-Methyl-D-Aspartate (NMDA) affected caffeine (0.6mM) neuroprotective effects against Aβ neurotoxicity in the SHSY5Y neuroblastoma cells. Data are the mean + standard error of four experiments and were analyzed using one-way ANOVA followed by Bonferroni test. * and ** shows the significance at 0.05 and 0.001 compared with the β-amyloid (Aβ) + caffeine (0.6mM)-treated group, respectively, and † is significance at 0.001 compared with the vehicle-treated group. Cell viability was assessed via MTT (3-[4,5-dimethylthiazol-2-yl]-2,5-diphenyl tetrazolium bromide) test.

**Figure 6:**
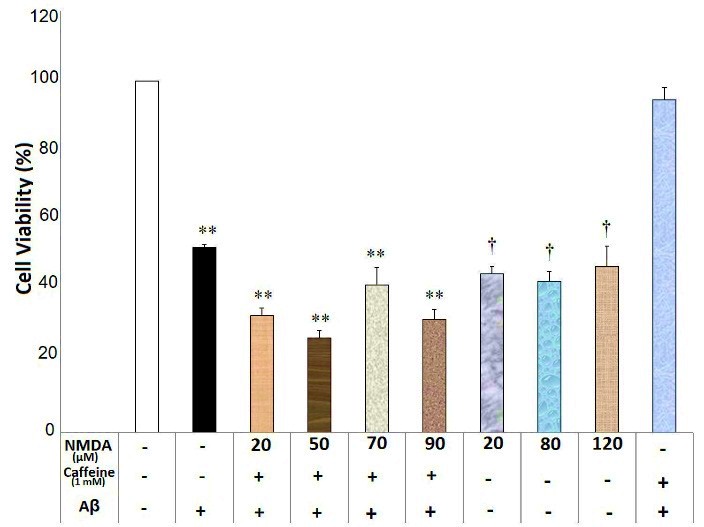
N-Methyl-D-Aspartate (NMDA) affected caffeine (1mM) neuroprotective effects against Aβ neurotoxicity in the SHSY5Y neuroblastoma cells. Data are the mean + standard error of four experiments and were analyzed using one-way ANOVA followed by Bonferroni test. ** shows the significance at 0.001 compared with the β-amyloid (Aβ) + caffeine (1mM)-treated group, and † is significance at 0.001 compared with the vehicle-treated group. Cell viability was assessed via MTT (3-[4,5-dimethylthiazol-2-yl]-2,5-diphenyl tetrazolium bromide) test.

NMDA at the concentrations of 20, 50, 70 and 90 μM inhibited caffeine (0.6 and 1mM) neuroprotective effects against Aβ neurotoxicity (F(6)=23.29, p=0.000 and F(6)= 42.95, p=0.000, respectively) (Figure 5 and 6). There was a significant difference regarding neuronal death between different concentrations of NMDA alone groups, Aβ-treated group and the vehicle treated group (F(3)= 0.657, p=0.594). Each concentration of NMDA (20, 80 and 120μM) significantly reduced neuronal viability compared with the vehicle-treated group (p<0.05) (Figure 5). However, there was no significant difference between NMDA alone groups and Aβ-treated group (p>0.05) (Figure 5).

Accumulating evidence confirms the idea that Aβ species have a prominent role in the pathophysiology of AD by inducing neurodegeneration and cognitive dysfunction.^18^ Thus, confronting Aβ–induced neural damage may be a hopeful strategy for curing this disorder or preventing disease progression.^18^ Our study, in line with previous ones, confirmed neuroprotective effects of caffeine against Aβ neurotoxicity in a neuronal cell line. In agreement, Dall'Igna et al showed that caffeine reduced Aβ neurotoxicity in the rat cerebellar neuronal culture^19^ and reversed Aβ-induced cognitive deficit in an animal model of AD.^20^ Epidemiologic studies have also revealed that the incidence of AD inversely correlated to the caffeine consumption.^17,21^ In contrast, some reports have shown that acute administration of caffeine may worsen neurological condition.^22^ Inconsistencies about neuroprotective effects of caffeine may mainly be related to the methodological differences in the studies.

Adenosine is an endogenous neuromodulator that influences many functions in the CNS.^23^ Adenosine fulfills homeostatic and neuromodulatory roles in the CNS by controlling neurotransmission and neuronal excitability.^24^ Although adenosine has four receptors (A_1_R, A_2A_R, A_2B_R, and A_3_R), most of its functions might be related to A_1_ and A_2A_ receptors.^24^ Adenosine and its receptors have important roles in the pathogenesis of neurodegenerative disorders like AD, though the exact function of this neuromodulator should be elucidated.^5^ It has been suggested that caffeine neuroprotective effects may be related to the inhibition of adenosine receptors.^20^ Our study showed that adenosine impeded neuroprotective effects of caffeine against Aβ neurotoxicity. Thus, the results of our study imply that the blockade of adenosine receptors may be responsible, at least partly, for the neuroprotective effects of caffeine in the cellular model of AD. In this regard, it has been shown that blockade of A_1_ and A_2A_ receptors produces neuroprotective effects.^19,20,22^ Our study showed that dantrolene, a ryanodine receptor antagonist,^25^ reduced neuroprotective effects of caffeine against Aβ neurotoxicity. Dantrolene modulates intracellular calcium by stabilizing and inhibiting RyR.^25^ It can be implicated that caffeine neuroprotective effect may contribute, at least in part, to the RyR-mediated calcium release from the endoplasmic reticulum. There are considerable controversies about the intracellular calcium and RyR roles in the pathogenesis of AD. In agreement with our results, systemic administration of dantrolene has increased amyloid plaque formation and deteriorated hippocampal neuronal damage in a transgenic model of AD.^26^ Accordingly, it has been proposed that increased RyR activity may attempt to maintain intracellular calcium homeostasis in the early stages of AD.^27^ In line with this, it has been shown that inability of neurons to up-regulate RyR3 made them more vulnerable to the insults like Aβ exposure, excitotoxicity, and oxidative stress.^28^ Thus, RyR hyperactivity in AD may be protective and/or a part of a mechanism to compensate calcium deregulation in AD.^27^ In contrast, short-term treatment with dantrolene has stabilized intracellular calcium, decreased amyloid load, and reversed cognitive decline and memory impairments in various AD mouse models.^29,30^

NMDA receptors are glutamate receptors with high permeability to calcium^6^ that have critical roles in synaptic plasticity, long-term potentiation and learning and memory.^31^ In contrast, hyperactivation of these receptors may lead to disrupting calcium homeostasis, neuronal damage, and excitotoxicity.^32^ Accumulating evidence has been shown that NMDA receptor-mediated excitotoxicity may be involved in the Aβ neurotoxicity.^33^ Our study showed that NMDA reduced caffeine neuroprotective effects against Aβ neurotoxicity. Thus, it can be implicated that caffeine neuroprotective effects may in part be related to the NMDA blockade. To best of our knowledge, there is very limited information in the literature about caffeine interaction with NMDA receptors. However, caffeine may mimic NMDA antagonist effects in some brain regions^34^ and its interaction with NMDA receptors may exert beneficial effects in neurodegenerative disorders.^35^ Thus, it is possible to assume that direct or indirect blockade of NMDA receptors may contribute to the caffeine neuroprotective effects.

### Limitations 

The main limitation of this study may be the administration of non-specific modulators of adenosine and RyR. Thus, using specific modulators of these receptors may help to further clarify exact mechanism of action of caffeine in managing neurodegenerative disorders.

## Conclusion

Caffeine produced neuroprotective effect against Aβ neurotoxicity in SHSY5Y cell line. The exact mechanism of caffeine neuroprotective effects is not completely clear. However, blockade of adenosine and NMDA receptors, as well as activation of RyR receptors, may contribute to the neuroprotective effects of caffeine. However, future studies with more selective and specific modulators of adenosine and RyR may confirm the results of this study. Moreover, we cannot rule out other mechanisms that may be involved in the neuroprotective effects of caffeine against Aβ neurotoxicity.

## Acknowledgments

We like to appreciate deputy for research of Shiraz University of Medical Sciences for financial support of this project.

## Ethical Issues

Not applicable

## Conflict of Interest

Non-declared

## References

[1] Selkoe DJ, Hardy J (2016). The amyloid hypothesis of alzheimer's disease at 25 years. EMBO Mol Med.

[2] Popugaeva E, Pchitskaya E, Bezprozvanny I (2017). Dysregulation of neuronal calcium homeostasis in alzheimer's disease - a therapeutic opportunity?. Biochem Biophys Res Commun.

[3] Chui DH, Tanahashi H, Ozawa K, Ikeda S, Checler F, Ueda O (1999). Transgenic mice with alzheimer presenilin 1 mutations show accelerated neurodegeneration without amyloid plaque formation. Nat Med.

[4] Berridge MJ, Bootman MD, Lipp P (1998). Calcium--a life and death signal. Nature.

[5] Gomes CV, Kaster MP, Tome AR, Agostinho PM, Cunha RA (2011). Adenosine receptors and brain diseases: Neuroprotection and neurodegeneration. Biochim Biophys Acta.

[6] Rozov A, Burnashev N (2016). Fast interaction between ampa and nmda receptors by intracellular calcium. Cell Calcium.

[7] Yamazawa T, Murayama T, Oyamada H, Suzuki J, Kurebayashi N, Kanemaru K (2016). Correlation of molecular dynamics analysis and calcium signaling in mutant ryanodine receptors. Biophys J.

[8] Danysz W, Parsons CG (2012). Alzheimer's disease, beta-amyloid, glutamate, nmda receptors and memantine--searching for the connections. Br J Pharmacol.

[9] Lacampagne A, Liu X, Reiken S, Bussiere R, Meli AC, Lauritzen I, et al. Post-translational remodeling of ryanodine receptor induces calcium leak leading to alzheimer's disease-like pathologies and cognitive deficits. Acta Neuropathol 2017. 10.1007/s00401-017-1733-7 28631094

[10] Woods LT, Ajit D, Camden JM, Erb L, Weisman GA (2016). Purinergic receptors as potential therapeutic targets in alzheimer's disease. Neuropharmacology.

[11] Fredholm BB, Battig K, Holmen J, Nehlig A, Zvartau EE (1999). Actions of caffeine in the brain with special reference to factors that contribute to its widespread use. Pharmacol Rev.

[12] McPherson PS, Kim YK, Valdivia H, Knudson CM, Takekura H, Franzini-Armstrong C (1991). The brain ryanodine receptor: A caffeine-sensitive calcium release channel. Neuron.

[13] Clark I, Landolt HP (2017). Coffee, caffeine, and sleep: A systematic review of epidemiological studies and randomized controlled trials. Sleep Med Rev.

[14] Liu J, Supnet C, Sun S, Zhang H, Good L, Popugaeva E (2014). The role of ryanodine receptor type 3 in a mouse model of alzheimer disease. Channels (Austin, Tex).

[15] Rosso A, Mossey J, Lippa CF (2008). Caffeine: Neuroprotective functions in cognition and alzheimer's disease. Am J Alzheimers Dis Other Demen.

[16] Kolahdouzan M, Hamadeh MJ (2017). The neuroprotective effects of caffeine in neurodegenerative diseases. CNS Neurosci Ther.

[17] Perez A, Li T, Hernandez S, Zhang R, Cao C (2016). The rationale of using coffee and melatonin as an alternative treatment for alzheimer’s disease. J Alzheimers Dis Parkinsonism.

[18] Ahmad A, Ali T, Park HY, Badshah H, Rehman SU, Kim MO (2017). Neuroprotective effect of fisetin against amyloid-beta-induced cognitive/synaptic dysfunction, neuroinflammation, and neurodegeneration in adult mice. Mol Neurobiol.

[19] Dall'Igna OP, Porciuncula LO, Souza DO, Cunha RA, Lara DR (2003). Neuroprotection by caffeine and adenosine a2a receptor blockade of beta-amyloid neurotoxicity. Br J Pharmacol.

[20] Dall'Igna OP, Fett P, Gomes MW, Souza DO, Cunha RA, Lara DR (2007). Caffeine and adenosine a(2a) receptor antagonists prevent beta-amyloid (25-35)-induced cognitive deficits in mice. Exp Neurol.

[21] Maia L, de Mendonca A (2002). Does caffeine intake protect from alzheimer's disease?. Eur J Neurol.

[22] de Mendonça A, Sebastião AM, Ribeiro JA (2000). Adenosine: Does it have a neuroprotective role after all?. Brain Res Rev.

[23] Martins IJ. Sirtuin 1 and adenosine in brain disorder therapy. J Clin Epigenet 2017;3(1).

[24] Fredholm BB, Chen JF, Cunha RA, Svenningsson P, Vaugeois JM (2005). Adenosine and brain function. Int Rev Neurobiol.

[25] Del Prete D, Checler F, Chami M (2014). Ryanodine receptors: Physiological function and deregulation in alzheimer disease. Mol Neurodegener.

[26] Zhang H, Sun S, Herreman A, De Strooper B, Bezprozvanny I (2010). Role of presenilins in neuronal calcium homeostasis. J Neurosci.

[27] Supnet C, Bezprozvanny I (2010). The dysregulation of intracellular calcium in alzheimer disease. Cell Calcium.

[28] Allan Butterfield D (2002). Amyloid β-peptide (1-42)-induced oxidative stress and neurotoxicity: Implications for neurodegeneration in alzheimer's disease brain. A review. Free Radic Res.

[29] Oules B, Del Prete D, Greco B, Zhang X, Lauritzen I, Sevalle J (2012). Ryanodine receptor blockade reduces amyloid-beta load and memory impairments in tg2576 mouse model of alzheimer disease. J Neurosci.

[30] Peng J, Liang G, Inan S, Wu Z, Joseph DJ, Meng Q (2012). Dantrolene ameliorates cognitive decline and neuropathology in alzheimer triple transgenic mice. Neurosci Lett.

[31] Morris RG, Anderson E, Lynch GS, Baudry M (1986). Selective impairment of learning and blockade of long-term potentiation by an n-methyl-d-aspartate receptor antagonist, ap5. Nature.

[32] Weilinger NL, Lohman AW, Rakai BD, Ma EM, Bialecki J, Maslieieva V (2016). Metabotropic nmda receptor signaling couples src family kinases to pannexin-1 during excitotoxicity. Nat Neurosci.

[33] Wang R, Reddy PH (2017). Role of glutamate and nmda receptors in alzheimer's disease. J Alzheimers Dis.

[34] Dall'Igna OP, Da Silva AL, Dietrich MO, Hoffmann A, de Oliveira RV, Souza DO (2003). Chronic treatment with caffeine blunts the hyperlocomotor but not cognitive effects of the n-methyl-d-aspartate receptor antagonist mk-801 in mice. Psychopharmacology (Berl).

[35] Brothers HM, Marchalant Y, Wenk GL (2010). Caffeine attenuates lipopolysaccharide-induced neuroinflammation. Neurosci Lett.

